# Revolutionizing protein degradation: Harnessing nanoparticles for PROTAC delivery

**DOI:** 10.1016/j.mtbio.2026.102949

**Published:** 2026-02-20

**Authors:** Yonghang Fan, Jianfen Su, Jun Yang, Xiaoling Guan, Yingjie Gong, Daliang Yang, Aiping Qin, Lingmin Zhang

**Affiliations:** a The Affiliated Panyu Central Hospital, Guangzhou Municipal and Guangdong Provincial Key Laboratory of Molecular Target & Clinical Pharmacology, the NMPA and State Key Laboratory of Respiratory Disease, Guangdong Basic Research Center of Excellence for Respiratory Medicine, School of Pharmaceutical Sciences, Guangzhou Medical University, Guangzhou, 511436, PR China; b The Second Affiliated Hospital, Guangzhou Medical University, Guangzhou, 510260, PR China; c Shenzhen Bao'an District Songgang People's Hospital, Shenzhen, 518000, PR China

**Keywords:** PROTAC, Nanoparticle delivery system, Drug delivery, Targeted protein degradation, Precision therapies

## Abstract

Proteolysis-targeting chimera (PROTAC) represents a paradigm shift in drug discovery, offering a promising therapeutic strategy for cancers, neurodegenerative diseases, and hematological malignancies. Unlike traditional small-molecule inhibitors that require occupancy of an active site, PROTAC operates via an event-driven mechanism, hijacking the ubiquitin-proteasome system to degrade target proteins. This approach can potentially convert “undruggable” targets into tractable ones, dramatically expanding the druggable proteome. However, the clinical translation of PROTAC faces a significant bottleneck due to inherent physicochemical and pharmacokinetic challenges, including poor solubility, limited membrane permeability, and off-target effects. Nanomedicine delivery systems have emerged as a powerful platform to overcome these hurdles. By encapsulating or conjugating PROTAC, nanocarriers can enhance bioavailability, improve cellular delivery, and increase target specificity, thereby unlocking their full therapeutic potential. This review systematically examines recent advances in nanoparticle-based PROTAC delivery. We illustrate how nanocarrier design—spanning organic, inorganic, biomimetic, and prodrug platforms—can optimize PROTAC properties and enhance therapeutic outcomes. Furthermore, we analyze current limitations and outline future directions to guide the development of next-generation delivery strategies, with the ultimate goal of accelerating the clinical translation of these transformative agents.

## Graphical abstract

**Figure:**
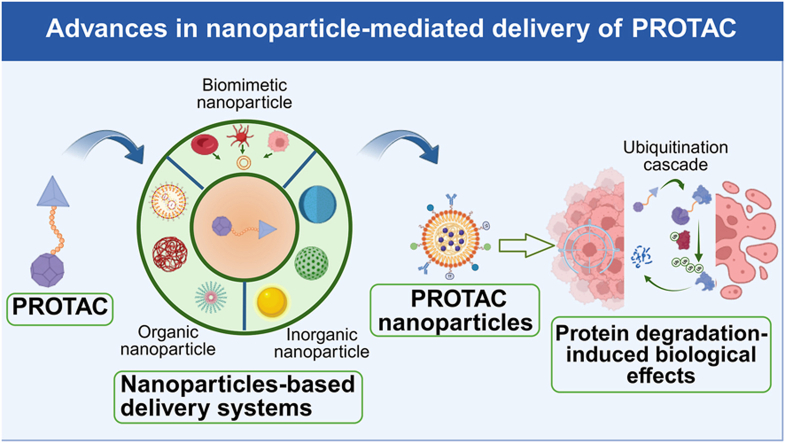

## 1 Introduction

The evolution from non-selective chemotherapy to targeted small-molecule inhibitors marked a paradigm shift in oncology. Despite their precision, these inhibitors are inherently limited by the need for a druggable binding site, excluding a vast majority of protein targets [1]. Proteolysis targeting chimera (PROTAC) molecules are novel agents prepared by targeted protein-degrading technology, which was first introduced by Craig M. Crews in 2001 [2]. PROTAC function by hijacking the ubiquitin-proteasome system (UPS)—which normally orchestrates fundamental cellular processes like apoptosis, gene regulation, and immunity by selectively degrading proteins—to induce target-specific ubiquitination and proteasomal degradation [3]. This mechanism shifts the pharmacological paradigm from occupancy-driven inhibition to event-driven degradation [4]. Traditional small-molecule inhibitors function through an occupancy-driven mechanism. This necessitates sustained high concentration at the target site and a well-defined, high-affinity binding pocket. Consequently, it excludes “undruggable” targets like transcription factors and scaffold proteins and is vulnerable to resistance mutations within the pocket [5]. By catalyzing the complete degradation of target proteins, PROTAC transcend these constraints and offer three fundamental advantages: access to an expanded target space, potent and sustained efficacy through catalytic, sub-stoichiometric action, and the capacity to overcome resistance by eliminating the target protein itself (Table 1) [6]. Early-phase clinical trials have reported objective responses in patients resistant to existing small-molecule therapies, suggesting the potential of this modality to target proteins previously considered “undruggable” [7].

**Table 1:** Small-molecule inhibitors and PROTAC drugs: mechanisms, targets, characteristics, and drug resistance.

\	small-molecule inhibitors	PROTAC drugs	Refences
Mechanisms	Static inhibition	Catalytic elimination	[[5], [6], [7]]
Characteristics	High dose	low dose, longer-lasting	
Targets	Proteins with druggable active-site pockets	Any protein containing a recognizable structured domain	
Drug resistance	Existence	Partially overcome	

**Fig. 1:**
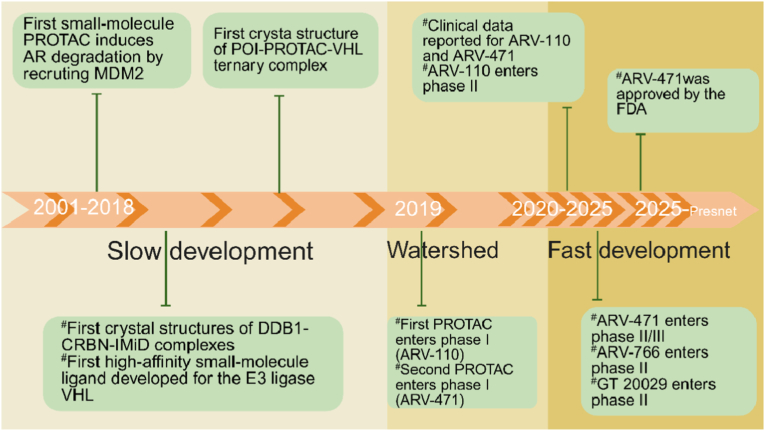
This timeline illustrates the major milestones in the evolution of PROTAC. PROTAC, proteolysis-targeting chimera; AR, androgen receptor; MDM2, mouse double minute 2 homolog; VHL, von‐Hippel–Lindau; DDB1, damage specific DNA binding protein 1; CRBN, cereblon; IMiD, Immune-mediated inflammatory disease; FDA, U.S. Food and Drug Administration. Reproduced with permission from Ref. [2]. Copyright © 2022, Springer Nature Limited.

This mechanistic leap—from static inhibition to catalytic elimination—has significantly advanced the field. Over the past two decades (Fig. 1) [2], translational teams have systematically converted the PROTAC concept into drug-like molecules, optimising linker geometry, ligase recruitment kinetics and oral bioavailability. Their cumulative effort has now delivered a clinical pipeline that embodies the promise: several promising PROTAC drugs have been researched because numerous researchers have dedicated their efforts to develop the PROTAC technology, which have progressed to clinical testing phases, including ARV-110 (targets androgen receptor (AR)), ARV-471 (targets estrogen receptor (ER)), and ARV-766 (targets ER).

PROTAC technology, rapidly developed over the past two decades, has demonstrated significant potential in treating various diseases, including cancers [8], neurodegenerative diseases [9] and malignant hematological disorders [10]. The therapeutic efficacy of PROTAC is achieved only after they reach the target cells and induce sufficient degradation of the target protein. However, formidable physiological barriers—the vascular endothelium, cell membranes, and lysosomes—combined with inherent PROTAC properties (high molecular weight, pronounced hydrophobicity, lack of tissue specificity) severely limit their delivery to disease sites and undermine therapeutic efficacy [11]. An effective delivery strategy is therefore critical. PROTAC delivery strategies are classified according to the mode of association between drug and carrier: biological vectors (utilizing natural or biomimetic transport systems), chemical conjugation, and physical encapsulation. Utilizing native or biomimetic carriers (such as exosome, cell), the biodelivery systems transport PROTAC to their targets while offering the inherent advantages of low immunogenicity, prolonged circulation, and precise targeting [12]. However, this method also faces several technical challenges. For instance, the clinical translation of exosomes, despite their biocompatible and targeted nature, poses two primary challenges: scalable production and storage standardization. Their generation remains inefficient, yielding mere milligrams from billions of cells at high cost [13]. Furthermore, the lack of defined storage protocols leads to particle aggregation, fusion, or functional loss, ultimately preventing reliable, large-scale manufacturing [14]. The chemical delivery strategy is characterized by the reversible derivatization of the PROTAC into a stable, masked precursor, enabling its targeted site-specific activation [15]. The prodrug approach represents the dominant chemical strategy for PROTAC delivery. Derivatization enhances solubility and pharmacokinetics, and the prodrug can be engineered for site-specific activation via passive or active targeting [16]. This strategy, however, faces several constraints: costly and complex bespoke synthesis, an intrinsic loading limit, stringent linker design requirements, and an increased molecular weight that may hinder tissue penetration [17]. As the mainstream strategy, physical delivery encompasses the use of exogenous nanocarriers that associate with PROTAC through hydrophobic, electrostatic, π–π stacking, or steric interactions, enabling encapsulation, dispersion, or surface loading [18]. This strategy enables modular, multifunctional integration. Nanoparticle delivery systems serve as the principal platform for realizing this strategy. Various types of nanoparticles have been explored for PROTAC delivery, including liposomal nanoparticles [19], polymeric nanoparticles [20], micellar nanoparticles [21], silica nanoparticles [22] and gold nanoparticles [23]. Nanoparticle delivery provides a crucial translational bridge for hydrophobic PROTAC. Encapsulation within lipid, polymeric, or inorganic cores enhances drug solubility and stability [24]. Surface PEGylation minimizes opsonization, prolonging circulation and promoting tumor accumulation via the EPR effect [25]. Further functionalization with stimuli-responsive (e.g., pH-, redox-, or enzyme-sensitive) components enables triggered payload releas [26]. Finally, constructing carriers from biodegradable materials (e.g., PLGA, CaP) ensures their clearance post-mission, fulfilling the essential efficacy-with-safety mandate [20].

Nanomaterials have advantages of enhancing drug stability and solubility, facilitating transmembrane transport, and prolonging circulation time, resulting in garnering extensive attention in the field of drug delivery [27]. Of course, they are facing several challenges including that they can induce immunogenicity in vivo and lead to rapid clearance. Moreover, they often show off-target effects in clinical applications because their active targeting capabilities are often limited.

Hybrid systems that integrate the ‘smart’ functions of biological components (e.g., targeting, evasion, penetration) with the ‘controllable’ features of synthetic nanomaterials (e.g., defined structure, high loading, tunable release) can overcome the limitations of conventional platforms. These systems are designed to achieve synergistic or emergent properties, combining biomimetic precision with industrial-grade designability and scalable production potential. Biomimetic nanoparticle, a typical hybrid system, is created by coating synthetic nanoparticles with natural cell membranes—a technique known as cell membrane coating. This biomimetic cloak serves as a protective layer, endowing the nanoparticles with enhanced stability, prolonged circulation, and improved targeting capabilities, representing a promising drug delivery strategy [28].

What's more, the deep integration of chemical and physical delivery paradigms also offers a path to transcend conventional nano-system limitations. By strategically modifying PROTAC into prodrugs that act as optimized building blocks, one can create self-assembling nanoparticles that circumvent exogenous carriers, thereby eliminating carrier-related immunogenicity and toxicity [29]. Furthermore, surface-engineered prodrug nanocarriers achieve long circulation and tumor enrichment, where disease-specific cues then trigger precise, site-selective activation for dose-sparing efficacy [30].

This review delineates how nanoparticle delivery can overcome the translational barriers facing PROTAC therapeutics. We first underscore the therapeutic advantages of catalytic degradation against the backdrop of key challenges—including high molecular weight, hydrophobicity, poor membrane permeability, and potential off-target effects—that collectively hinder efficient delivery. Subsequent sections dissect the design, recent progress, and outstanding gaps in lipid-, polymer-, and inorganic-based nanoplatforms, while charting their convergence with biomimetic engineering and chemical prodrug strategies. The review concludes with a survey of the clinical landscape and a proposed roadmap for next-generation, intelligent delivery systems to accelerate the clinical translation of PROTAC.

## 2 PROTAC technology: event-driven degradation and the delivery challenge

**Fig. 2:**
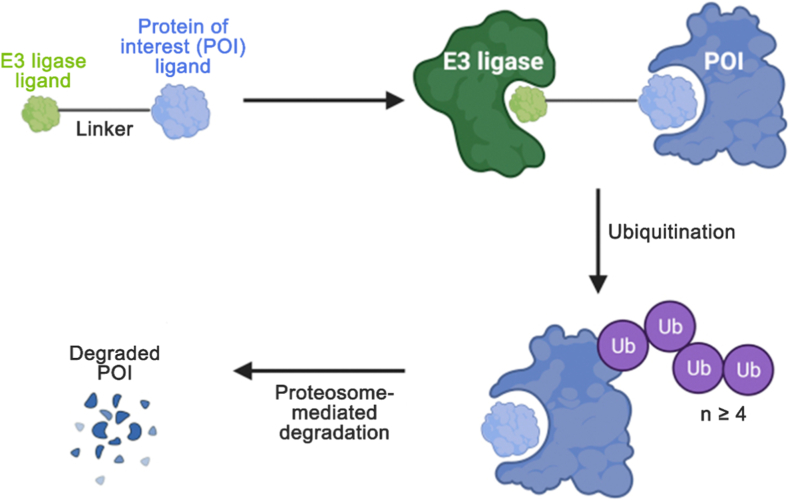
Mechanism of action of PROTAC drugs. Reproduced with permission from Ref. [31]. Under the CC BY-NC-ND license.

PROTAC transcend classical inhibitors by a catalytic degradation mechanism. The catalytic mode of action operates through a four-step cycle (Fig. 2): (1) formation of a ternary complex with the target protein and an E3 ligase, (2) polyubiquitination of the target, (3) its proteasomal degradation, and (4) release of the PROTAC for recycling [4,5]. Thus, its utility stems from three core advantages. First, they target ligandable surfaces, not just deep pockets, unlocking “undruggable” target classes [32]. Second, contrary to stoichiometric inhibitors, their sub-stoichiometric action delivers durable effects with low exposure [33]. Third, selectivity is enhanced by the cooperative formation of a target-PROTAC-ligase ternary complex [34].

Despite its potentiality, PROTAC can also face the multitude of significant challenges in clinical applications. On one hand, PROTAC is deficient in aqueous solubility and cellular permeability because it typically has a molecular weight greater than 700 Da and exhibits high surface polarity, resulting in reducing the drug administration within the biological system, and further preventing it from reaching the effective therapeutic concentration required to exert its pharmacological effects [35].

On the other hand, PROTAC is protein-specific degraders with limited tissue specificity. Although the human genome encodes over 600 E3 ubiquitin ligases, most PROTAC is based on only two E3 ligases: cereblon (CRBN) and von Hippel-Lindau (VHL) tumor suppressor, which are widely expressed in most human tissues and may contribute to cause off-target or accumulate in non-target tissues [36]. Exploring less ubiquitous, tissue- or context-restricted E3 ligases could enable the development of degrader drugs with inherent spatial control, thereby potentially minimizing systemic toxicity. Recent expansion of the ligandable E3 ligase toolbox has provided a new chemical foundation for next-generation PROTAC. Key advances include: Pei et al. identify piperlongumine as a ligand for X-linked inhibitor of apoptosis (XIAP) to achieve Cyclin dependent kinase 9 (CDK9) degradation [37]; Huang et al. demonstrate that the diterpenoid oridonin covalently recruits Ring finger protein 114 (RNF114) [38]; and Poirson et al. discover Kelch domain containing 2 (KLHDC2) as a small-molecule-recruitable ligase [39]. These discoveries have significantly expanded the repertoire of E3 ligases available for targeted protein degradation. In addition to rapid systemic clearance [2], PROTAC can face another major intracellular barrier: entrapment within endosomes and subsequent degradation in lysosomes, which severely limits their cytosolic bioavailability [40]. Therefore, it is necessary to seek strategies that enhance efficacy while minimizing toxicity.

## 3 Application of nanoparticle-based PROTAC delivery

Nanomaterials for PROTAC delivery are broadly categorized as organic or inorganic nanoparticles. Each class possesses distinct design principles, physicochemical properties, and biological behaviors, providing a versatile toolkit to address specific delivery bottlenecks.

### 3.1 Advances in organic nanoparticle-based PROTAC delivery

Organic nanoparticles are solid particles composed of natural or synthetic organic molecules, typically with diameters ranging from 10 to 100 nm in dynamic light scattering (DLS) [41]. These nanoparticles are often constructed by proteins, lipids, polymers, and carbohydrates, which confers important properties such as high aqueous solubility, ease of cellular uptake, non-toxicity, and biodegradability. These characteristics make organic nanoparticles highly suitable for various biomedical applications [42].

#### 3.1.1 Advances in liposome nanoparticle-based PROTAC delivery

**Fig. 3:**
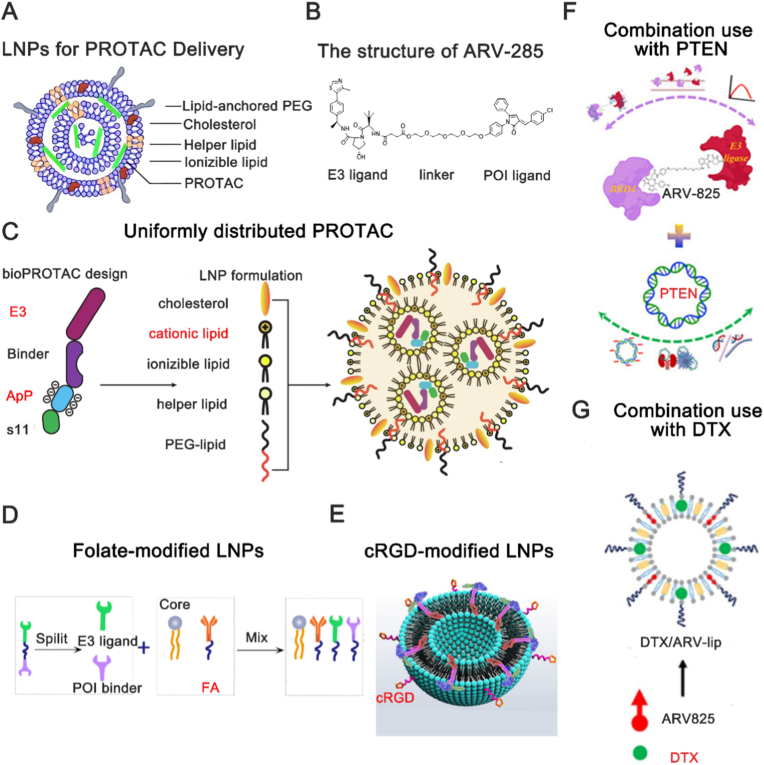
Applications of lipid nanoparticles (LNPs) in PROTAC drug delivery. (A) Representative LNPs structure for PROTAC delivery. PEG, polyethylene glycol. Reproduced with permission from Ref. [43]. Under CC BY 4.0 license. (B) The structure of ARV-285. POI, point of interest. Reproduced with permission from Ref. [44]. Under CC BY 4.0 license. (C) Purified bioPROTAC include an E3 ligase, binding domain, ApP, and GFP s11 tag. The fusion proteins are formulated as LNPs including ionizable/cationic lipids, neutral helper lipids, and lipid-anchored PEG. PEG, polyethylene glycol. Reproduced with permission from Ref. [45]. Under CC BY 4.0 license. (D) Schematic diagram of the novel split-and-mix liposome PROTAC platform and the schematic diagram of FA targeted protein degradation. FA, folate. Reproduced with permission from Ref. [46]. Copyright © 2023 American Chemical Society. (E) Construction of the STAT3-degrading liposomal PROTAC prodrug. cRGD, cyclic Arg-Gly-Asp. Reproduced with permission from Ref. [47]. Copyright © 2024 American Chemical Society. (F) Synergistic combination of PTEN plasmid and BRD4 PROTAC-loaded lipid nanocarriers. PTEN, phosphatase and tensin homolog. Reproduced with permission from Ref. [48]. Under CC BY 4.0 license. (G) Preparation of DTX and ARV825 co-loaded LNPs. DTX, docetaxel. Reproduced with permission from Ref. [49]. Copyright © 2024 Elsevier B.V.

Liposome nanoparticles (LNPs) represent one of the most mature and highly investigated strategies in drug delivery. Composed primarily of two basic components—phospholipids and cholesterol—LNPs leverage the amphiphilic nature of phospholipids, which possess a polar head and a nonpolar tail. This molecular structure enables the spontaneous formation of bilayer vesicles when hydrated above their critical micelle concentration (Fig. 3A). When PROTAC drugs (Fig. 3B) are encapsulated within LNPs in an aqueous medium, the LNPs form spherical micelles (ranging in size from 0.05 to 5.00 μm, including small unilamellar vesicles (SUV, 20-80 nm) and multilamellar vesicles (MLV, >200 nm)) with a hydrophilic exterior and a hydrophobic interior. This configuration enhances the solubility of PROTAC drugs and facilitates their transport across physiological barriers and cellular membranes through mechanisms such as endocytosis, translocation, and fusion with the cell membrane [50]. These features ensure the successful delivery of PROTAC drugs into the biological system and their subsequent uptake by cells. LNPs have been employed for the delivery of individual PROTAC drugs. To streamline delivery, Chen et al. encapsulated a pre-conjugated E3P–PROTAC within LNPs. This strategy facilitates rapid ternary complex assembly at the target protein, improving cellular permeability, avoiding the hook effect, and speeding up degradation [51]. The uniform distribution of PROTAC drugs within LNPs can improve therapeutic efficacy. Chan et al. used a “bioPROTAC” by replacing the small-molecule E3 ligand of a conventional PROTAC with the full E3 enzyme itself. To enable efficient encapsulation, they incorporated an anionic ApP ligand, ensuring uniform distribution within cationic LNPs (Fig. 3C). This yielded a stable, ready-to-use formulation that activates degradation on demand, significantly extending PROTAC functionality [45]. Functional optimization of LNPs is a promising approach to enhance the delivery of PROTAC drugs, improving specific delivery, cellular uptake by target cells, and reducing off-target effects [52]. LNPs can be surface-decorated with ligands such as carbohydrate ligands [53], folic acid ligands [54], and Arg-Gly-Asp (RGD) peptide ligands [55]. Song et al. separated the E3 ligase receptor and target protein receptor of SM-PROTAC drugs and self-assembled them with the effective components of LNPs and folic acid (Fig. 3D) [46]. This approach effectively addressed the issue of low drug efficacy associated with peptide-based SM-PROTAC. The study demonstrated that folate-decorated PROTAC drugs exhibited significant tissue targeting. Wang et al. used cyclic Arg-Gly-Asp (cRGD)-modified cationic LNPs to encapsulate PROTAC prodrugs (Fig. 3E) [47]. This method not only ensured high tissue targeting but also facilitated lysosomal escape, thereby reducing drug loss. The efficacy of a single drug has its limitations. To more effectively curb disease progression, LNPs-mediated co-delivery of multiple drugs is considered a promising strategy. By simultaneously delivering a PROTAC drug with other molecules, therapeutic efficacy can be enhanced or side effects can be reduced from a disease treatment perspective. Fu et al. employed polyethylene glycol modificated (PEGylated) LNPs to co-deliver bromodomain protein 4 (BRD4)-targeting PROTAC (ARV) and Ni [44], and Saraswat et al. used nano- LNPs to deliver phosphatase and tensin homolog (PTEN) plasmid and ARV (Fig. 3F) [48]. Huang et al. encapsulated BRD4-PROTAC and a photosensitizer within LNPs [56]. Photosensitizers, such as macrocyclic organic compounds or engineered dyes, generate cytotoxic reactive oxygen species upon light irradiation [57]. All these studies demonstrated enhanced therapeutic efficacy. Additionally, LNPs have been used as adjuvants for PROTAC delivery. Chen et al. utilized LNPs as a co-delivery vehicle for ARV825 and docetaxel (DTX) (Fig. 3G) [49]. ARV825 downregulated the expression of proteins in cancer cells that confer resistance to DTX, thereby enhancing the antitumor efficacy of DTX without adverse side effects. Overall, liposome nanoparticles (LNPs) for PROTAC delivery exhibit high bioavailability, strong loading capacity, and sustained release characteristics [58]. Compared with other nanoparticle carrier systems, LNPs have proven to be highly successful in terms of safety and efficiency for PROTAC delivery [45]. However, several unavoidable drawbacks exist in LNP-mediated PROTAC delivery. First, long-term administration of LNPs can inevitably trigger immune responses within the body, leading to reduced drug efficacy or inducing toxic side effects. Second, the complex preparation process of LNPs, which requires precise control conditions and expensive equipment, results in time-consuming and costly production with limited yield, restricting their application in large-scale manufacturing. Finally, LNPs can produce the lipid layer rupture and drug leakage during storage due to the poor stability, leading to drug inactivation.

#### 3.1.2 Advances in polymer nanoparticle-based PROTAC drug delivery

**Fig. 4:**
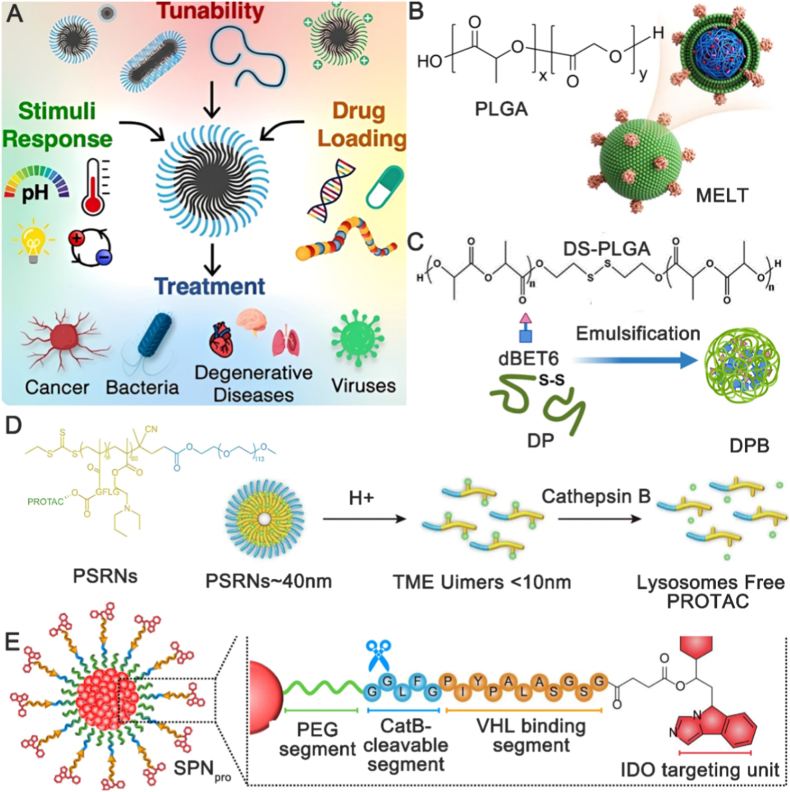
Applications of polymer nanoparticles in PROTAC drug delivery. (A) Polymeric nanoparticles for drug delivery. Reproduced with permission from Ref. [62]. Under CC BY 4.0 license. (B) PLGA-mediated delivery of dTRIM24. PLGA, poly(lactic-co-glycolic acid); MELT, M2 macrophage membrane/PLGA//dTRIM24. Reproduced with permission from Ref. [63]. Copyright © 2023, The Author(s), under exclusive licence to Shanghai Institute of Materia Medica, Chinese Academy of Sciences and Chinese Pharmacological Society. (C) DS-PLGA-mediated delivery of dBET6. DS-PLGA, PLGA-S-S-PLGA; DP, DS-PLGA; DPB, dBET6/DS-PLGA. Reproduced with permission from Ref. [64]. Under CC BY 4.0 license. (D) PSRNs-mediated delivery of CDK4/6-targeting PROTAC. TME, tumor microenvironment; PROTAC, proteolysis-targeting chimeras; PSRN, pH/cathepsin B dual-responsive nanoparticle platform. Reproduced with permission from Ref. [65]. Under CC BY 4.0 license. (E) SPN _pro_-mediated delivery of PROTAC drugs. PEG, polyethylene glycol; VHL, von‐Hippel–Lindau; IDO, indoleamine-pyrrole 2,3-dioxygenase; CatB, cathepsin B. Reproduced with permission from Ref. [66]. Under CC BY 4.0 license.

Researchers have increasingly played their attention to other nanomaterials for PROTAC drug delivery due to the inherent limitations of LNPs, such as polymer nanoparticles (PNPs), which have advantages of well-established fabrication processes and widespread applications. PNPs are a macromolecule composed of many repeating monomer units. For example, hydrophilic monomers like N-vinylpyrrolidone (NVP) impart surface hydration, enhancing colloidal stability [59]; degradable monomers such as lactide (LA) improve long-term biocompatibility, thus avoiding the accumulation risks associated with non-degradable materials [60]; ionic/environment-responsive monomers like dimethylaminoethyl (DMAEMA) methacrylate protonate in acidic endosomes/lysosomes, thereby generating a strong proton-sponge effect that facilitates cytosolic escape [61]. These can be produced by the simple and cost-effective methods compared to LNPs, including self-assembly and adsorption. A polymer backbone, linkers, targeting moieties, and the drug payload are the main component of PNPs (Fig. 4A). The linker is a crucial component in polymer nanoparticles, as it facilitates drug loading and release [67]. There are two primary mechanisms for drug release from polymer nanoparticles: external stimuli and internal stimuli. External stimuli involve using controllable factors such as ultrasound, temperature, and radiation to achieve site-specific drug release. In contrast, internal stimuli leverage the unique intracellular environment of cancer cells (e.g., slightly acidic pH (pH ≤ 6.5), elevated levels of reductants, and/or enzymes) to trigger drug release [68]. PNPs that degrade in response to elevated levels of reductants in the cancer cell environment have been utilized for PROTAC drug delivery. Liu et al. utilized redox-responsive poly (disulfide amide) polymeric (PDSA) to construct ARV@PDSA Nano-PROTAC [69], which significantly enhanced drug accumulation in the tumor microenvironment. Leveraging the slightly acidic pH of the tumor microenvironment is one of the most common strategies for polymer nanoparticle-based PROTAC delivery. Huang et al. exploited the degradation properties of poly (lactic-co-glycolic acid) (PLGA) under acidic conditions to successfully deliver dTRIM24 to the target site (Fig. 4B) [63]. Combining multiple stimuli for PROTAC delivery has shown greater potential in polymer nanoparticles. To address the partial degradation of PLGA and the potential escape of intact or damaged nanoparticles from lysosomes during endocytosis [70], improved PLGA nanoparticles with enhanced delivery capabilities have been developed. Guan et al. modified PLGA by incorporating disulfide bonds responsive to pH and glutathione (GSH), creating a dual-responsive poly (lactic-co-glycolic acid) (DS-PLGA) (Fig. 4C) [64]. This modification demonstrated superior drug delivery efficiency. Yang et al. developed a pH/cathepsin B dual-responsive nanoparticle platform (PSRN) (Fig. 4D) [65], which not only enhanced PROTAC delivery but also effectively avoided lysosomal sequestration, further promoting drug accumulation. Combining polymer nanoparticle-based PROTAC delivery with immunotherapy has shown enhanced therapeutic efficacy in disease treatment. Wang et al. demonstrated that semiconductor polymer nanoPROTAC combined with immunotherapy could effectively suppress the growth of both subcutaneous and deep-seated tumours covered by tissues [71]. Zhang et al. further showed that the combination of nanoPROTAC and immunotherapy could also serve as a phototheranostic component (Fig. 4E), which allows to detect the biodistribution, tumor targeting, and drug release upon excitation at specific wavelengths [66]. In another study, the same team targeted prostaglandin E2 (PGE2) using a similar approach, achieving comparable therapeutic effects [72]. Despite the improvements in PROTAC drug delivery provided by polymer nanoparticles, there are still two major limitations. First, polymer nanoparticles may have insufficient biocompatibility. As foreign materials, they can interact with biological components, potentially triggering immune or inflammatory responses. Second, although the fabrication process for producing polymer nanoparticles is well-established, there also remains some technical challenges how to keep nanoparticles in ideal size and shape to optimize PROTAC delivery. Additionally, there is a demanding task to achieve stable conjugation of PROTAC drugs with polymer nanoparticle. It is necessary to focus on enhancing the biocompatibility of polymer nanoparticles, improving fabrication techniques, and considering individual differences to promote the widespread clinical application of PROTAC drugs.

#### 3.1.3 Advances in polymer micelle-based PROTAC drug delivery

**Fig. 5:**
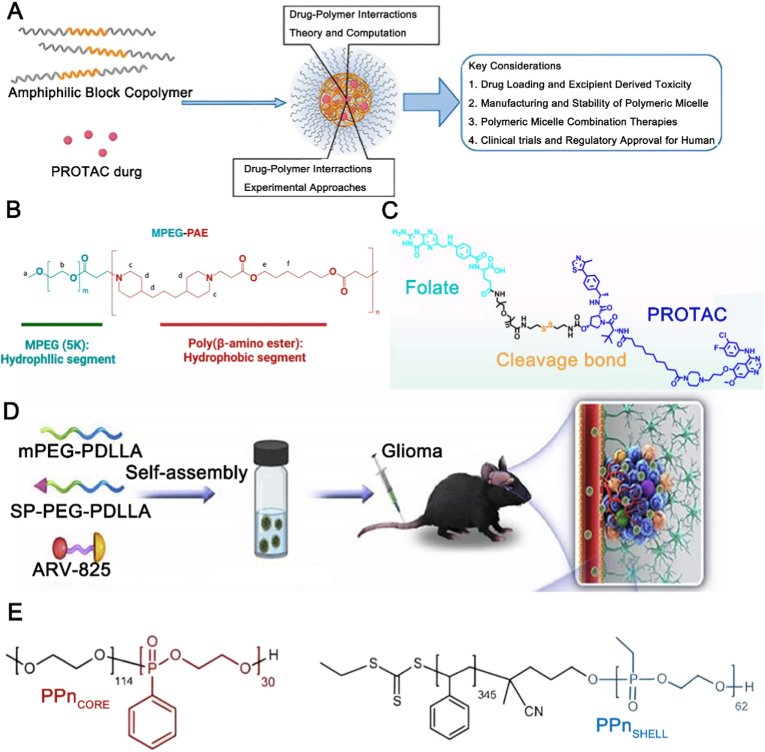
Applications of PMs-based PROTAC delivery. (A) The typical core-shell structure and advantages of PMs. Reproduced with permission from Ref. [73]. Under CC BY 4.0 license. (B) Application of PMs-mediated PROTAC delivery in transdermal patches. PEG_5K_, polyethylene glycol 5000. Reproduced with permission from Ref. [21]. Copyright © 2023 American Chemical Society. (C) Self-assembled PMs for PROTAC delivery. Reproduced with permission from Ref. [74]. Copyright © 1999-2025 John Wiley & Sons, Inc or related companies. (D) PMs-mediated delivery of ARV-825. mPEG-PDLLA, methoxy poly (ethylene glycol)-poly (d,l-lactic acid); SP-PEG-PDLLA, substance P peptide-modified poly (ethylene glycol)-poly (d,l-lactic acid). Reproduced with permission from Ref. [75]. Under the CC BY-NC-ND license. (E) PPn-based PMs for PROTAC delivery. PPn, polyphosphonates. Reproduced with permission from Ref. [76]. Under CC BY 4.0 license.

Given the respective limitations of LNPs and PNPs for PROTAC delivery, research efforts are directed toward developing a hybrid material that integrates their advantages while overcoming their inherent drawbacks. Polymer micelles (PMs) have emerged as a promising alternative, combining advantages over both LNPs and polymeric nanoparticles. Compared to LNPs, which may degrade or fuse in vivo causing drug leakage, PMs offer superior stability and better payload protection. Furthermore, PMs typically exhibit reduced immune recognition and clearance relative to many polymeric nanoparticles, thereby enhancing their biocompatibility and potential for prolonged circulation. PMs, particularly those based on PEG-PLGA amphiphiles, self-assemble into core-shell nanostructures (Fig. 5A) [77]. In this configuration, PROTAC is hydrophobically sequestered within the PLGA core, while the PEG corona provides steric stabilization [78]. The corona forms a hydration shell that minimizes protein adsorption and immune clearance, thereby prolonging systemic circulation and enabling passive tumor targeting via the enhanced permeability and retention (EPR) effect [79]. Current types of PMs include amphiphilic block copolymers, graft polymer micelles, amphiphilic random copolymers, polyelectrolyte micelles, and smart polymer micelle delivery systems [80]. Smart, or stimuli-responsive, drug release represents a key advantage of PMs. Chen et al. developed pH-responsive MPEG–poly (β-amino ester) micelles that undergo rapid, acid-triggered disintegration, releasing their PROTAC payload in acidic endosomes and the tumor microenvironment (Fig. 5B) [21]. In vivo, this stimulus-responsive release led to significantly greater tumor suppression compared to non-responsive controls, validating acidity-triggered liberation as an effective strategy to enhance PROTAC bioavailability and antitumor efficacy. PMs have been engineered as “drug–carrier-in-one” systems. For instance, Ma et al. conjugated a folate-PEG (FA-PEG) moiety to a PROTAC via a tumor-abundant, glutathione-cleavable disulfide linker, creating an FA-PEG-PROTAC conjugate. This design integrates folate-receptor-mediated targeting, PEG-mediated long circulation, and cytosolic release via disulfide cleavage, which collectively enhanced antitumor efficacy in EGFR-overexpressing models (Fig. 5C) [74]. Customized targeting significantly broadens PMs applications. By engineering Angiopep-2-decorated PEG-PDLLA micelles encapsulating ARV-825 (Fig. 5D), Yang et al. achieved blood-brain barrier penetration and glioma-targeted delivery—a strategy that successfully extends PROTAC-based treatment to complex central nervous system cancers [75]. Toward unified theranostic platforms, Koshkina et al. engineered polyphosphazene (PPn) micelles that serve a dual role: their tuned ^31^P chemistry grants high MRI relaxivity for imaging, while their hydrophobic core loads PROTAC for therapy (Fig. 5E). This integration of carrier and contrast agent permits real-time, non-invasive monitoring of tumor accumulation, which can guide therapeutic dosing [76]. Collectively, PMs has matured into a multidimensional toolbox. As demonstrated, Chen et al. exploited acidic pH for burst release; Ma et al. achieved superior delivery efficiency by designing a unified drug–carrier conjugate; Yang et al. employed ligand-receptor targeting to traverse the blood-brain barrier; and Koshkina et al. fused materials chemistry with MRI to enable theranostics. Though divergent in design, these platforms are unified by a common logic: each senses a specific disease cue to achieve precise, on-demand PROTAC delivery and an enhanced therapeutic index. In summary, polymeric micelles provide an integrated platform for PROTAC delivery, combining solubilization, targeting, and stimuli-responsive release. Advancing these systems toward the clinic will require expanded chemical libraries, rational stability design, and clinically predictive evaluation.

### 3.2 Advances in inorganic nanoparticle-based PROTAC delivery

There are also certain inherent limitations to organic nanoparticles in PROTAC drug delivery despite the advantages, which they can make a certain matter difficult to remain stable under harsh conditions such as high temperature or high pressure due to their poor chemical stability. Additionally, it is challenging to improve drug loading because their drug-loading capacity is limited. In light of these drawbacks, inorganic nanoparticles have garnered attention in PROTAC drug delivery when they exhibit strong stability in biological environments [81]. Additionally, inorganic nanoparticles have made the attractive candidates for PROTAC delivery in advantages of the high surface reactivity, the substantial drug-loading capacity, and the controlled drug-release capabilities [82] such as gold and silica nanoparticles [83].

#### 3.2.1 Advances in silica nanoparticle-based PROTAC drug delivery

**Fig. 6:**
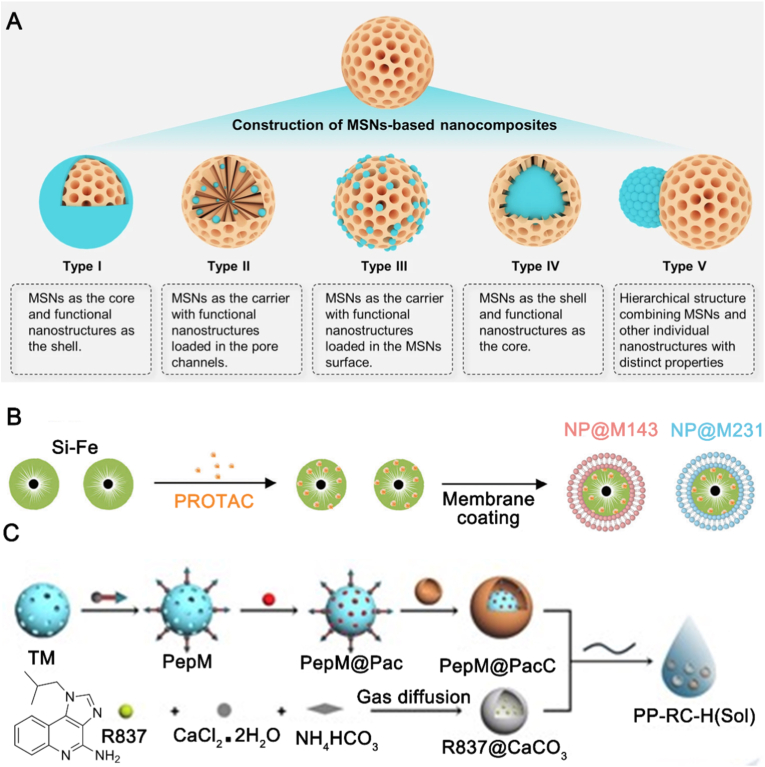
Applications of silica nanoparticles in PROTAC drug delivery. (A) Various nanostructured MSNs-based nanocomposites. MSNs, mesoporous silica nanoparticles. Reproduced with permission from Ref. [84]. Under CC BY 4.0 license. (B) Hybrid silica nanoparticles for PROTAC delivery. Reproduced with permission from Ref. [85]. Copyright © 1999-2025 John Wiley & Sons, Inc or related companies. (C) Silica nanoparticle composites for PROTAC delivery. Reproduced with permission from Ref. [20]. Copyright © 1999-2025 John Wiley & Sons, Inc or related companies.

Silica nanoparticles possess a honeycomb-like structure with hollow channels such as mesoporous silica nanoparticles (MSNs), making them highly promising for drug delivery applications (Fig. 6A) [86]. MSNs are often modified to be surface-responsive by these modifications called “gatekeepers” to ensure the successful release of PROTAC drugs. Gatekeepers that bind to the exterior of the mesoporous silica pores can prevent the release of PROTAC drugs until an external stimulus is applied, resulting in drug release [87]. Common gatekeepers include stimulus-responsive polymers or polysaccharides, such as chitosan [88]. Poly-L-lysine, a photo-stimuli-responsive polymer, has been applied in silica nanoparticle-mediated PROTAC delivery. Zhan et al. constructed a photo-responsive silica nanoparticle using poly-L-lysine to deliver dBET6 (termed USDPR, a near-infrared light-controlled PROTAC delivery device) [89]. Upon near-infrared (NIR) light stimulation, poly-L-lysine degrades, opening the channels within the silica nanoparticles and allowing the PROTAC drug to reach the target site, effectively degrading BRD4. When silica nanoparticles are combined with other inorganic nanoparticles, they can compensate for the limitations of single-component silica nanoparticles in drug delivery. Hybrid silica nanoparticles composed of silica and another inorganic material have been used for PROTAC drug delivery. Tan et al. encapsulated PROTAC within self-tumor cell membrane-coated silica-iron oxide hybrid nanoparticles (Fig. 6B), achieving targeted drug delivery [85]. Additionally, the contrast agent properties of iron oxide nanoparticles enabled real-time monitoring of antitumor treatment efficacy, facilitating timely adjustments to the therapeutic regimen. Composite nanoparticles including silica and other inorganic material have also been used for PROTAC delivery. Wu et al. linked MSNs coated with cell membranes to CaCO_3_ nanoparticles loaded with R837 (a TLR7/8 agonist, an imidazoquinoline amine analog of guanosine) using a thermo-sensitive PLGA-PEG-PLGA copolymer (Fig. 6C), achieving dual effects of immunotherapy and chemotherapy [20].

#### 3.2.2 Advances in gold nanoparticle-based PROTAC drug delivery

**Fig. 7:**
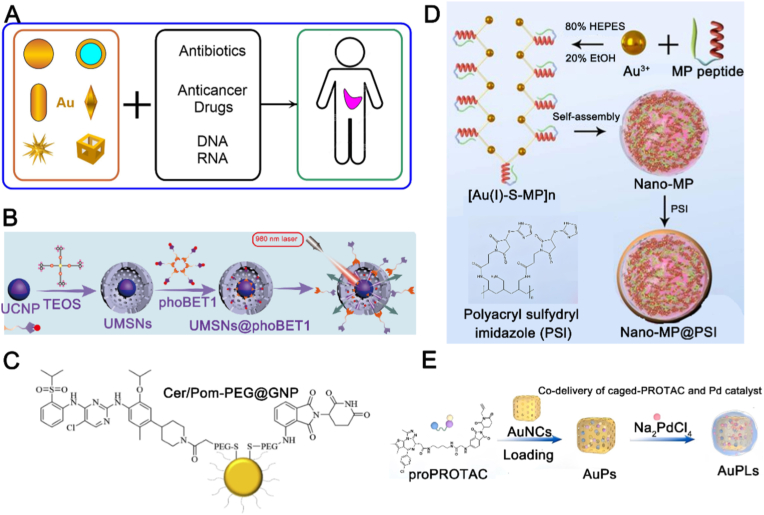
Applications of gold nanoparticles in PROTAC drug delivery. (A) Various gold nanoparticles can be used as drug carriers for targeted therapy. Reproduced with permission from Ref. [90]. Under CC BY 4.0 license. (B) Co-delivery of PROTAC using GNPs combined with silica nanoparticles. UCNP, upconversion nanoparticle; TEOS, tetraethyl orthosilicat. Reproduced with permission from Ref. [22]. Copyright © 2023 American Chemical Society. (C) Covalent delivery of PROTAC on GNPs. Reproduced with permission from Ref. [91]. Copyright © 2020 Elsevier B.V. (D) pH-responsive GNPs for PROTAC delivery. MP, MDMX predator; HEPES, N-2-hydroxyethylpiperazine-N-2-ethane sulfonic acid; EtOH, ethyl alcohol. Reproduced with permission from Ref. [23]. Under the terms of the Creative Commons Attribution License. (E) Co-delivery of PROTAC using GNPs for expanded therapeutic applications. Reproduced with permission from Ref. [92]. Copyright © 2024 Elsevier B.V. (For interpretation of the references to colour in this figure legend, the reader is referred to the Web version of this article.)

Gold nanoparticles (GNPs) have emerged as a complementary approach to silica nanoparticles, addressing some of their limitations and enhancing the delivery of PROTAC drugs (Fig. 7A). He et al. encapsulated PROTAC drugs within a core-shell structure composed of GNPs and a silica nanocoating (Fig. 7B), which significantly increased drug delivery to the target site and inhibited tumor growth [22]. GNPs offer several advantages in drug delivery, including their unique physicochemical properties and versatile surface chemistry [93]. Two primary loading strategies are used for delivering PROTAC drugs with GNPs: covalent and non-covalent conjugation. Covalent conjugation has been widely explored. Wang et al. successfully delivered PROTAC drugs by covalently linking them to GNPs via S-Au bonds (Fig. 7C) [91]. To enhance tumor accumulation of PROTAC drugs, Yan et al. synthesized a pH-responsive macromolecule called poly (sulfonated imidazole) (PSI) to coat Nano-MPs (Fig. 7D), which demonstrated potent anticancer activity while maintaining good safety and favorable in vivo clearance properties [23]. Non-covalent conjugation avoids altering the drug's properties, which can be an issue with covalent bonding. However, due to its weaker binding, drug molecules may easily dissociate from the GNPs surface, limiting its use in drug delivery [83]. GNPs have also been used for the co-delivery of PROTAC prodrugs. Li et al. developed a gold-nanocube-based platform (AuPLs) for on-demand PROTAC delivery. The system encapsulates both a PROTAC prodrug and a palladium catalyst within a temperature-sensitive phase-change layer, which is stable at body temperature. Upon near-infrared irradiation, the photothermal effect melts the layer, co-releasing the prodrug and catalyst. Subsequently, the catalyst activates the PROTAC in situ via bioorthogonal chemistry. This photothermally triggered, catalytically activated design showcases the sophisticated spatiotemporal targeting achievable with gold nanostructures (Fig. 7E) [92].

Research on inorganic nanoparticles for PROTAC drug delivery remains relatively limited. The biosafety of inorganic nanomaterials is also an important consideration, as some inorganic nanoparticles (such as GNPs and MSNs) are difficult to degrade in vivo and may accumulate in the liver and spleen for prolonged periods. In addition, similar to unmodified organic nanoparticles, inorganic nanomaterials also exhibit certain drawbacks, such as non-biodegradability, low biocompatibility, and short circulation times when unmodified, which restrict their applications [94].

### 3.3 Advances in biomimetic nanoparticle-based PROTAC delivery

**Fig. 8:**
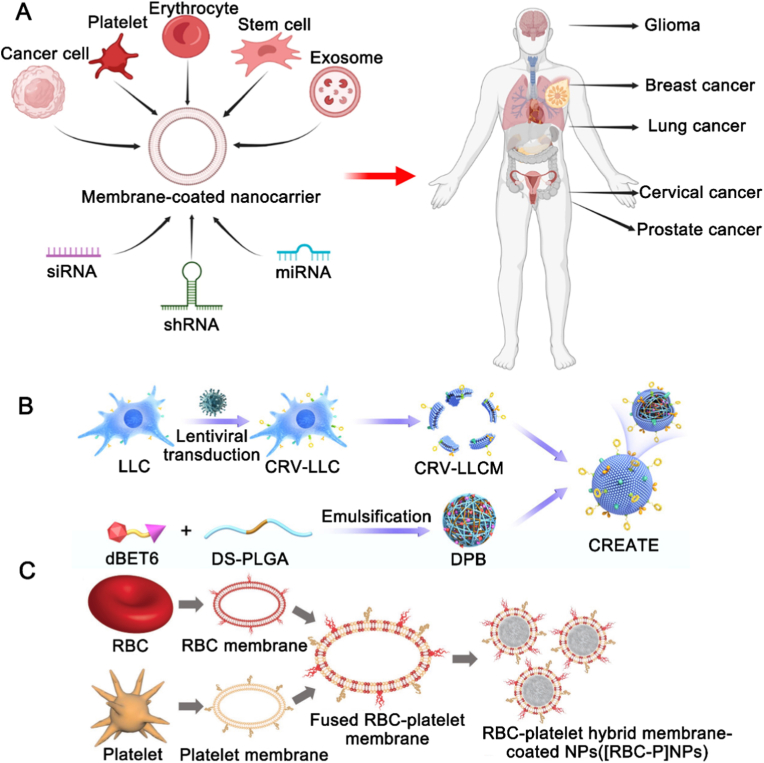
Applications of biomimetic nanoparticles in PROTAC drug delivery. (A) Classification of biomimetic nanoparticles. Reproduced with permission from Ref. [100]. Copyright © 2023 Elsevier Ltd. (B) Cancer cell membrane-coated biomimetic nanoparticles for PROTAC delivery. LLC, Lewis lung carcinoma; CRV-LLC, CRV‐engineered Lewis lung carcinoma; CRV-LLCM, CRV‐engineered Lewis lung carcinoma cell membranes; CREATE, CRV‐LLC membrane/DS‐PLGA/dBET6. Reproduced with permission from Ref. [101]. Copyright © 1999-2025 John Wiley & Sons, Inc or related companies. (C) Hybrid membrane-coated biomimetic nanoparticles for PROTAC delivery. RBC, red blood cell. Reproduced with permission from Ref. [102]. Copyright © 1999-2025 John Wiley & Sons, Inc or related companies. (For interpretation of the references to colour in this figure legend, the reader is referred to the Web version of this article.)

Biomimetic technologies that inherit the intrinsic features of native cells, such as cell membrane coating, have emerged as important means to address these limitations, offering high biocompatibility and prolonged circulation in vivo. Additionally, due to the complex composition of biological membranes, which contain both “self-recognition molecules” and “self-labeling molecules,” biomimetic nanoparticles can evade the immune system and specifically deliver molecules to target proteins [95]. Common biological membranes used in biomimetic nanotechnology include stem cell membranes [96], macrophage membranes [97], red blood cell membranes [98], and cancer cell membranes [99] (Fig. 8A). Cancer cell membrane-coated nanoparticles have been used for PROTAC drug delivery. Zhang et al. successfully designed a novel biomimetic nanoparticle drug delivery system (Fig. 8B) for targeting pancreatic cancer cells, demonstrating excellent homotypic targeting ability as well as good biocompatibility and immunocompatibility [101]. Huang et al. effectively utilized macrophage membranes for PROTAC delivery, modulating macrophage phenotypes to treat atherosclerosis [63]. Beyond single-cell membranes, combinations of different cell membranes can be engineered to leverage their specific functions for enhanced delivery efficiency [103]. Hybrid cell membrane-coated nanoparticles have also been explored for PROTAC delivery. Dehaini et al. fabricated platelet-red blood cell hybrid membranes to cloak nanoparticles (Fig. 8C), retaining protein markers from each source cell and combining their unique functionalities [102]. These hybrid membranes offer superior targeting, biocompatibility, and immune regulation capabilities. Despite the significant potential of biomimetic nanotechnology for PROTAC delivery, challenges such as high manufacturing costs and difficulties in blood acquisition have restricted its application to laboratory settings, limiting large-scale production.

### 3.4 Advances in nanoparticle-based PROTAC prodrug delivery

**Fig. 9:**
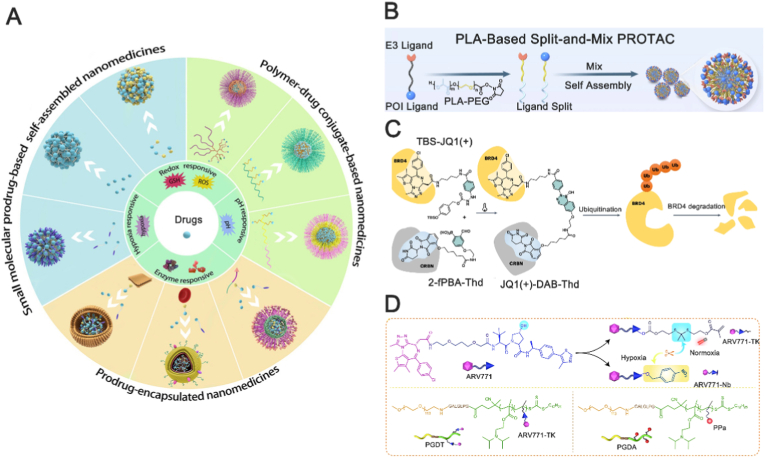
Applications of nanoparticles in PROTAC prodrug delivery. (A) Nanocarriers for prodrug delivery. Reproduced with permission from Ref. [106]. Under the CC BY-NC-ND license. (B) The delivery strategy of split-and-mix PROTAC. PLA, poly (lactic acid). Reproduced with permission from Ref. [107]. Under the CC BY-NC-ND license. (C) Schematic representation of in situ synthesized JQ1(+) DAB-Thd recruiting CRBN to degrade BRD4. ‘Thd’ stands for thalidomide and targets CRBN. Reproduced with permission from Ref. [88]. Under the CC BY-NC-ND license. (D) Structure of the ROS/hypoxia dual-activatable PROTAC nanoparticle (PGDAT@N). PPa, photosensitizer pyropheophorbide-a. Reproduced with permission from Ref. [29]. Under the CC BY-NC-ND license.

The past decade has witnessed an exponential surge in PROTAC (proteolysis-targeting chimera) nanomedicines, propelled by the conceptual elegance of event-driven protein degradation. Yet, as we transition from “target-everything” exuberance to registrational dossiers, a recurrent Achilles’ heel—affinity-limited drug leakage—is emerging as a non-trivial translational liability [104]. Because PROTAC operate catalytically (sub-stoichiometric, multiple turnover), even nanomolar “leak” can saturate E3 ligases in healthy tissues, producing on-target protein degradation in the wrong cell type [105]. The prodrug‐within‐nanocarrier architecture (Fig. 9A) converts the prototypical PROTAC payload into a catalytically silent, low‐molecular‐weight construct that is selectively unmasked within the pathological microenvironment. By appending metabolically labile, yet sterically compact, masking modules, the chimeric molecule exhibits three orders of magnitude lower binary–ternary complex propensity in off-target tissues, while retaining a enzyme-mediated cleavage at the disease site. A sterically compact and electron-withdrawing pro-moiety simultaneously elevates chemical stability and aqueous solubility of the cloaked PROTAC without compromising log D at the tumour site [108]. Finally, the prodrug–PROTAC as a molecular amphiphile can quantitatively self-assemble into monodisperse nanoparticles without recourse to auxiliary excipients, eliminating the affinity-limited leakage typically observed when parent PROTAC is physically loaded into pre-formed carriers. Thereby, it converts the erstwhile “leakage liability” into a self-shielding, self-targeting delivery modality [109]. Emerging PROTAC–prodrug nanoarchitectures are converging on three orthogonal and mechanistically complementary trajectories: (i) “Divide-to-Assemble” delivery strategy, (ii) Bio-orthogonal click delivery strategy, (iii) Conditionally activated prodrugs delivery strategy [8,110]. “Divide-to-Assemble” delivery strategy reconceptualises the PROTAC pharmacophore as a pair of “complementary, self-recognising monomers”, which each rendered catalytically void by the absence of the counterpart. The monomers are chemically tuned to display orthogonal π-stacking and hydrogen-bond codes that remain cryptic under physiological ionic strength, yet undergo “entropy-driven co-assembly” when confronted with the pathological extracellular matrix signature [111]. Building on the divide-to-assemble concept, Zhan et al. [107] leveraged poly (lactic acid) (PLA)—an FDA-affirmed, clinically de-risked aliphatic polyester—to engineer PLA-PEG-based SM-PROTAC micelles (Fig. 9B). PLA-PEGylation yields lower-molecular-weight precursors with superior membrane permeability. The resulting nanoparticles concomitant with a markedly extended systemic half-life and exhibit superior stability, robust tumour growth inhibition and a prolonged therapeutic window composed to the benchmark PROTAC ARV-825. The bio-orthogonal click delivery strategy exploits azide–alkyne cycloaddition to pair two inert precursors inside tumours, forging a covalent bond that instantly generates active PROTAC in situ [112]. This delivery modality guarantees precursor stability in circulation and thereby enhances drug safety. Wang et al. [113] devised a “decaging-to-ligation” (D2L) platform activated by the phenylalanine analogue Phe-BF_3_ (Fig. 9C). The PROTAC prodrug comprises TBS-JQ1(+) and 2-fPBA-Thd. Upon robust uptake of Phe-BF_3_ by target cells, the boronic acid selectively cleaves the TBS group, unmasking JQ1 that immediately condenses with 2-fPBA-Thd to form a boron-containing five-membered heterocycle. The resulting in situ assembly recruits the E3 ligase and efficiently degrades BRD4, affording precise spatiotemporal drug delivery. Conditionally activated prodrugs delivery strategy—currently the most widely adopted paradigm—entails reversible derivatization of the PROTAC into a cleavable pro-form that is grafted onto a nanocarrier and subsequently released only within a defined pathological milieu [114]. Site-specific release is routinely engineered to sense either intrinsic tumour hallmarks—ROS, enzymes, GSH, hypoxia—or extrinsic cues such as light or X-rays. Gao et al. [8] appended ROS-cleavable thioketal (TK) or hypoxia-labile 4-nitrobenzyl linkers to the BRD4-directed PROTAC ARV-771 (Fig. 9D). Exploiting elevated MMP-2 and low pH within the tumour milieu, and amplifying local ROS by the photosensitizer pyropheophorbide-a (PPa), the construct achieved intracellularly confined drug liberation. This not only eradicated established tumour cells but also eradicated their nascent pool, preventing tumourigenesis at its origin. Zou et al. [29] conjugated a PROTAC prodrug to clinically approved PEG-b-PLGA via a disulfide spacer; intracellular glutathione triggered re-activation. Co-assembly with doxorubicide yielded nanoparticles that lowered the MDA-MB-231 IC_50_ to 0.036 μM, exemplifying synergistic PROTAC-chemo combination therapy.

### 3.5 The clinical translation of PROTAC: current landscape and the role of nanotechnology

The clinical advancement of ARV-110 in 2019, as the first PROTAC candidate, bolstered confidence in the therapeutic potential of this modality. This pivotal step was followed by increased domestic development, leading to the clinical entry of agents like Haisco's HSK29116 (a bruton tyrosine kinase (BTK) degrader) and Kintor's GT20029 (an AR degrader) in 2021. This activity is complemented by a growing pipeline of other PROTAC molecules currently in clinical trials (Table 2).

**Table 2:** Selected PROTAC drugs in clinical trials.

Drug Name	Clinical Trial Phase	E3 ligases	target proteins	target diseases	Route of Administration	Clinical Trial Results
ARV-471 (Vepdegestrant)	Phase III	VHL or CRBN	ER	Breast cancer	oral	Exhibited sustained antitumor activity in patients with multiply relapsed disease.
NX-2127	Phase I	CRBN	BTK	B-cell malignancies	oral	Overcoming BTK inhibitor resistance
ARV-110 (Bavdegalutamide)	Phase II	CRBN	AR	Prostate cancer	oral	Therapeutic efficacy depends on biomarker status
KT-474/SAR444656	Phase II	CRBN	IRAK4	Inflammatory diseases	oral	The first PROTAC to reach clinical trials for a non-oncological indication
CFT7455	Phase I/II	CRBN	IKZF1, IKZF3	Relapsed/refractory multiple myeloma	i.v.	Intravenous administration

Analysis of the clinical pipeline reveals three emerging trends: (1) a shift toward oral bioavailability, (2) the expansion into non-oncological areas such as inflammatory diseases, and (3) a focus on overcoming resistance to established therapies. However, limitations such as poor oral bioavailability and rapid metabolism constrain their clinical utility. Nanoparticles fulfill key roles in advancing PROTAC by: (1) enhancing solubility and stability for reliable parenteral administration, (2) potentiating targeted, on-demand release to maximize efficacy and safety, (3) promoting tumour penetration and homogeneous drug distribution, and (4) permitting theranostic integration. Ultimately, the clinical success of PROTAC may hinge on the synergistic development of the degrader molecule alongside its engineered nanocarrier.

## 4 Challenges and prospects of nanoparticle-based PROTAC delivery

Nanotechnology offers a promising solution to these challenges, potentially expanding the therapeutic applicability of PROTAC. What's more, nanomedicine systems markedly attenuate the lysosomal drug clearance. Conventionally, internalized drugs traverse early endosomes, late endosomes, and lysosomes, where degradation and elimination curtail efficacy. Nanoparticle platforms circumvent this fate by promoting endosomal escape: ionizable lipids become protonated within acidic endosomes, triggering membrane rupture or a proton-sponge effect that releases the payload into the cytosol and potentiates therapeutic action [62]. The “proton-sponge” effect is characteristic of polycationic nanocarriers such as polyethylenimine (PEI) and polyamidoamine (PAMAM) dendrimers. This mechanism involves the buffering of protons by surface amines within the acidified lysosome. To maintain the pH gradient, the V-ATPase pump is driven to import more H^+^ ions, accompanied by an influx of Cl^−^ ions and water. The resulting osmotic swelling ultimately leads to lysosomal membrane rupture and cytosolic release of the cargo [[115], [116], [117]]. In contrast, the membrane fusion strategy utilizes pH-sensitive lipids or polymer. The acidic environment triggers a structural change—such as a lamellar-to-hexagonal phase transition or a major polymer conformational shift—that either mediates fusion with or causes destabilization of the lysosomal membrane. This allows for the direct discharge of the PROTAC payload into the cytoplasm [118,119].

While the goal is to develop safer, more effective protein-degradation therapies by fully leveraging nanoparticles, a number of hurdles must be overcome. First, the inevitable immunogenicity of nanoparticles poses a critical issue. As an external substance, the delivery of PROTAC via nanoparticles has the potential to induce both innate and adaptive immunogenicity. Innate responses occur upon first exposure, triggered by the surface charge or hydrophobicity of LNPs or polymeric micelles, which activates the complement cascade. Finally, the therapeutic efficacy and safety will be reduced after macrophages can trigger a rapid increase, leading to immune responses, inflammation, or allergic reactions. Repeated administration induces acquired immunogenicity, most notably via the generation of anti-PEG antibodies in response to PEGylated materials. This accelerated clearance of subsequent doses [120]. Second, while the biodegradable nature of many experimental nanomaterials suggests safety, their precise breakdown processes remain unclear. This uncertainty, coupled with the risk of long-term accumulation, raises valid concerns about potential off-target toxicity to normal cells. Further research into biocompatibility and improved degradation technologies is needed to mitigate these concerns. Despite these challenges, the application of nanotechnology in PROTAC drug delivery offers substantial promise (Table 3).

**Table 3:** The landscape of nanocarriers in PROTAC delivery: pportunities and challenges.

Delivery system type	Materials/Specific form	PROTAC-loaded (target)	Advantages	Limitations	References
Organic Nanoparticle	Liposome	ARV-771 ( BET), ARV825 (BRD4)	1 A membrane-like structure that facilitates fusion or interaction 2 Simple preparation process 3 Co-encapsulated both hydrophilic and lipophilic compounds within a single nanocarrier	1 Poor organ-specific targeting 2 Low endosomal escape efficiency	[29,40]
	polymer	ARV-771 (BET), dTRIM24 (TRIM24)	1 Structure controllability 2 Biodegradability 3 Variable drug delivery capability	1 Particle aggregation 2 Protein corona formation	[45,51]
	Polymer Micelle	ARV825 (BRD4), EGFR-targeting PROTAC			[58,74]
Inorganic Nanoparticle	Silica	dBET6 (BRD4), focal adhesion kinase (FAK-P) targeting PROTAC	1 Unique external field responsiveness 2 Rigid structure 3 Powerful diagnostic and imaging capabilities	1 Non-degradability and accumulation 2 Heavy metal ion release	[63,121]
	Gold	Androgen receptor splice variant‐7 (AR‐V7) targeting PROTAC, BRD4 targeting PROTAC			[92,122]
	CaP	-	1 Degradation products can directly participate in the body's mineral metabolism cycle 2 Excellent ‘endosomal escape’ capability 3 pH-responsive dissolution	1 Limited types of drug carriers 2 Poor stability	-
Biomimetic Nanoparticle	Cell membrane encapsulation, exosomes	dBET6 (BRD4), dTRIM24 (TRIM24)	1 Organizational homing ability 2 Long cycle time 3 Low immunogenicity	1 Low drug loading 2 Difficult directional engineering transformation	[80,101]
Molecular Engineering Strategy	PROTAC prodrug	MZ1-O (BRD4), dBET1 (BRD4)	1 Precise molecular design 2 Multiple targeting 3 No carrier-related toxicity	1 Complex chemical synthesis 2 A specific drug structure is required	[123,124]
	PROTAC conjugates	ARV-771 (BRD4), MS432 (MEK), MS99 (ALK)			[125]

The next generation of nanodelivery systems should embody the principles of intelligence, efficiency, and safety, guided by the following priorities (1) employing biodegradable materials (e.g., specific inorganic ions or polymers): with well-defined metabolic pathways over persistent carriers like silica or non-erodible metals [126]; (2) achieving multi-tier targeting, from tissue-level specificity down to subcellular organelles [127]; (3) ensuring high-efficiency loading (via covalent or host-guest chemistry) coupled with reliable on-demand release at the target site [128]; (4) adopting immunocooperative designs—capable of evading immune detection when necessary or stimulating it for synergy [129]; (5) incorporating early assessment of scalable manufacturing to ensure viable clinical and commercial translation.

Intrinsically biodegradable metal oxides can overcome the inherent non-degradability of conventional inorganic PROTAC nanocarrier [130]. For instance, ZnO nanoparticles dissolve into Zn^2+^ ions within acidic compartments (e.g., tumours or endolysosomes), directly linking carrier disintegration to PROTAC release. The resulting Zn^2+^ ions can additionally induce mitochondrial apoptosis, providing a synergistic therapeutic outcome [131]. Similarly, calcium-based inorganic nanomaterials represent a readily biodegradable and biocompatible alternative [132]. Calcium phosphate (CaP) matrices undergo pH-dependent dissolution. The released ions help neutralize lysosomal acidity and destabilize the membrane, thereby enhancing cytosolic delivery. Crucially, Ca^2+^ is endogenous electrolytes that undergo efficient renal and gastrointestinal clearance, substantially reducing the risk of long-term tissue accumulation [133].

PROTAC conjugates, encompassing ligand-, antibody-, aptamer-, and peptide-linked variants, represent a complementary strategy for precision delivery [134]. In this approach, the conjugate acts as a targeting warhead that specifically homes to disease markers, while the nanocarrier serves as a versatile delivery vehicle that ferries and shields the payload until its release at the target site [135]. Early proof-of-concept studies underscore this synergistic potential: By conjugating a PROTAC to a ROR1-specific monoclonal antibody, Wang et al. [136] achieved enhanced tumor targeting and amplified apoptotic response compared to ligand-based delivery alone. Zhang et al. [137] overcame lapatinib resistance in HER2+/PIK3CA-mutant breast cancer by tethering the PROTAC to the PI3K pan-inhibitor copanlisib. Utilizing a peptide-PROTAC conjugate, Wang et al. [138] investigated the glucose-metabolic axis connecting FOXM1 to PD-L1 expression.

As a hybrid modality combining a new molecular entity with a novel delivery system, PROTAC–nanomedicines face a uniquely complex regulatory pathway. Their development necessitates precise definition and control of critical quality attributes, requires comprehensive safety assessment of both carrier and drug—individually and in combination—and demands early, science-driven dialogue with regulatory agencies to establish fit-for-purpose standards.

Overall, applying the nanoparticle delivery technology in PROTAC drugs enhances the potential to transform “undruggable” targets into “druggable” ones. The rapid development of nanotechnology in PROTAC drug research also brings hope for treating many challenging diseases.

## 5 Conclusions

In summary, PROTAC technology offers a revolutionary, catalytic strategy to target “undruggable” proteins and overcome drug resistance. However, its clinical translation is gated by inherent pharmacological challenges, including poor solubility, low membrane permeability, and inadequate tissue specificity. Nanoparticle-based delivery systems have emerged as a pivotal solution to these bottlenecks.

This review has systematically examined the design principles, advances, and limitations of nanocarriers for PROTAC, encompassing organic, inorganic, biomimetic, and prodrug-based platforms. Organic nanoparticles (e.g., LNPs, PNPs) excel in biocompatibility and ease of functionalization. Inorganic carriers (e.g., MSNs, GNPs) provide high drug-loading capacity and unique physical properties. Biomimetic nanotechnologies leverage natural design for enhanced targeting and immune evasion. Nano-prodrug strategies enable spatiotemporally controlled activation, minimizing off-target release and improving therapeutic safety.

While nanotechnology has markedly improved the delivery efficiency and specificity of PROTAC, challenges remain regarding immunogenicity and long-term material safety. Future research must focus on developing biodegradable, smart-responsive nanoplatforms capable of cooperative immune modulation, and on the deep integration of chemical, physical, and biological delivery paradigms. Success in this endeavor will be crucial for transforming PROTAC from potent molecular tools into next-generation therapeutics that deliver tangible clinical benefit.

## Compliance with ethics requirements

This article does not contain any studies with human or animal subjects.

## CRediT authorship contribution statement

**Yonghang Fan:** Data curation, Investigation, Methodology, Software, Validation. **Jianfen Su:** Investigation, Methodology, Software, Writing – original draft. **Jun Yang:** Conceptualization, Resources, Supervision, Writing – original draft. **Xiaoling Guan:** Formal analysis, Investigation, Methodology, Software. **Yingjie Gong:** Methodology, Software. **Daliang Yang:** Conceptualization, Resources, Writing – original draft. **Aiping Qin:** Funding acquisition, Resources, Supervision, Writing – original draft, Writing – review & editing. **Lingmin Zhang:** Conceptualization, Funding acquisition, Project administration, Resources, Supervision, Writing – original draft, Writing – review & editing.

## Declaration of competing interest

The authors declare that they have no known competing financial interests or personal relationships that could have appeared to influence the work reported in this paper.

## Figures and Tables

**Table:** 

PROTAC	Proteolysis targeting chimera
UPS	Ubiquitin-proteasome system
AR	Androgen receptor
MDM2	Mouse double minute 2 homolog
VHL	Von‐Hippel–Lindau
DDB1	Damage specific DNA binding protein 1
CRBN	Cereblon
IMiD	Immune-mediated inflammatory disease
FDA	U.S. Food and Drug Administration
XIAP	X-linked inhibitor of apoptosis
CDK9	Cyclin dependent kinase 9
RNF114	Ring finger protein 114
KLHDC2	Kelch domain containing 2
DLS	Dynamic Light Scattering
LNPs	Liposome nanoparticles
SUV	Small unilamellar vesicles
MLV	Multilamellar vesicles
RGD	Arg-Gly-Asp
cRGD	cyclic Arg-Gly-Asp
PEGylated	Polyethylene glycol modificated
BRD4	Bromodomain protein 4
PTEN	Phosphatase and tensin homolog
DTX	Docetaxel
PEG	Polyethylene Glycol
POI	point of interest
FA	Folate
PNPs	Polymer nanoparticles
NVP	N-vinylpyrrolidone
LA	Lactide
DMAEMA	Dimethylaminoethyl
PDSA	Poly (disulfide amide) polymeric
PLGA	Poly (lactic-co-glycolic acid)
GSH	Glutathione
DS-PLGA	Dual-responsive poly (lactic-co-glycolic acid)
PSRN	pH/cathepsin B dual-responsive nanoparticle platform
PGE2	Prostaglandin E2
MELT	M2 macrophage membrane/PLGA//dTRIM24
DP	DS-PLGA
DPB	dBET6/DS-PLGA
TEM	Tumor microenvironment
IDO	Indoleamine-pyrrole 2,3-dioxygenase
Cat B	cathepsin B
PMs	Polymer micelles
EPR	Enhanced permeability and retention
PPn	Polyphosphazene
PEG_5K_	Polyethylene Glycol 5000
mPEG-PDLLA	Methoxy poly(ethylene glycol)-poly(d,l-lactic acid)
SP-PEG PDLLA	Substance P peptide-modified poly (ethylene glycol)-poly (d,l-lactic acid)
MSNs	Mesoporous silica nanoparticles
NIR	Near-infrared
GNPs	Gold nanoparticles
PSI	Poly (sulfonated imidazole)
UCNP	Upconversion nanoparticle
TEOS	Tetraethyl orthosilicate
MP	MDMX predator
HEPES	N-2-Hydroxyethylpiperazine-N-2-Ethane Sulfonic Acid
EtOH	Ethyl Alcohol
LLC	Lewis lung carcinoma
CRV-LLC	CRV‐engineered Lewis lung carcinoma
CRV-LLCM	CRV‐engineered Lewis lung carcinoma cell membranes
CREATE	CRV‐LLC membrane/DS‐PLGA/dBET6
RBC	Red blood cell
PLA	Poly (lactic acid)
PPa	Photosensitizer pyropheophorbide-a
oral	Administering oral medications
i.v.	Intravenous injection
PEI	Polyethylenimine
CaP	Calcium phosphate
BET	Bromodomain and extra terminal domain
TRIM24	Tripartite motif containing 24
MEK	Mitogen-activated extracellular signal-regulated kinase

## Data Availability

Data will be made available on request.
